# Morphology of male genitalia, legs, and wing venation reveals the classification of Mesozoic Zoraptera (Insecta)

**DOI:** 10.1186/s12983-025-00595-x

**Published:** 2026-02-07

**Authors:** Petr Kočárek, Ivona Kočárková, Robin Kundrata

**Affiliations:** 1 Department of Biology and Ecology, Faculty of Science, University of Ostrava, Chittussiho 10, 710 00 Ostrava, Czech Republic; 2 Department of Zoology, Faculty of Science, Palacky University, 17. Listopadu 50, 771 46 Olomouc, Czech Republic

**Keywords:** Fossils, Amber, Systematic paleontology, Taxonomy, Cretaceous, Polyneoptera, Insects

## Abstract

The current classification of extant Zoraptera is based on the results of molecular phylogeny combined with the morphology of male genitalia and supplemented by the characters on the male abdomen and the number of metatibial spurs. However, fossil representatives of Zoraptera have not yet been classified into the modern system, and most of them remain in the catch-all genus *Zorotypus* Silvestri, 1913, because the genitalia are either not observable or have not been examined in detail. In this study, for the first time, we describe and critically evaluate the male genitalia and other principal diagnostic characters of all available Mesozoic Zoraptera. Our results have led to the first proposal of a generic classification of Mesozoic Zoraptera. We describe two new genera, *Cretozoros* gen. nov. and *Burmazoros* gen. nov., reinstate *Paleospinosus* Kaddumi, 2005, stat. restit. from synonymy with the subgenus *Octozoros* Engel, 2003 (in *Zorotypus*), and elevate *Octozoros* Engel, 2003 to the genus level. *Cretozoros* gen. nov., *Paleospinosus* Kaddumi, 2005, stat. restit., and *Octozoros* stat. nov. are classified in Spiralizoridae: Latinozorinae, whereas *Burmazoros* gen. nov. and *Xenozorotypus* Engel & Grimaldi, 2002 are classified in Zorotypidae: Zorotypinae. Altogether, it was possible to classify nine of the 11 currently recognized species of Mesozoic Zoraptera. To evaluate the phylogenetic relationships of fossil zorapterans, including the newly established taxa, we estimated the evolutionary histories of selected morphological features using ancestral character state reconstruction. The classification of Mesozoic Zoraptera within the modern system enables us to better understand the early evolution of this insect order.

**Supplementary Information:**

The online version contains supplementary material available at 10.1186/s12983-025-00595-x.

## Introduction

Zoraptera represents one of the smallest insect orders, with only 47 extant and 16 extinct described species [1–5]. Members of Zoraptera are small, soft-bodied, and primarily winged polyneopteran insects. However, winged specimens are rare, and the majority of collected specimens are wingless. Wing dimorphism is one of a few autapomorphies of the order and is correlated with the presence or absence of compound eyes and ocelli and the presence or absence of distinct pigmentation, with alate specimens being distinctly darker [6]. Zorapterans are known predominantly from the subtropics and tropics, where they typically inhabit the bark of rotting wood. Although zorapterans may be common insects in the tropics, they have rarely been collected because of their cryptic lifestyle and inconspicuous appearance [7].

Zoraptera represents an ancient evolutionary lineage with a Paleozoic origin [8–10]. Although Misof et al. [11] suggested that Zoraptera diverged from a common ancestor shared with Dermaptera in the Middle Jurassic (ca 180–160 Ma), later analyses [10] placed the split as early as 370 Ma. Most findings suggest that the origin of Zoraptera dates to the Paleozoic and that they were already diversified and widely distributed in Gondwana at the time of the supercontinent’s fragmentation [1, 12].

Uniformity in the general morphology of Zoraptera has led to the persistence of a conservative classification of extant species, with only a single nominotypical genus, *Zorotypus* Silvestri, 1913, in the family Zorotypidae for more than a century [6]. Kočárek et al. [13] and Matsumura et al. [9] conducted molecular phylogenetic studies using a combination of nuclear and mitochondrial markers. These independent analyses revealed two major phylogenetic lineages, which Kočárek et al. [13] classified as families Zorotypidae and Spiralizoridae, each of which was further subdivided into two robustly supported subfamilies [13]. The updated classification was supported by synapomorphies in the structure and shape of the male genitalia and other taxonomically valuable characteristics, including the morphology of the apex of the male abdomen and the number of spurs on the metatibia. Unfortunately, molecular genetic methods cannot be applied to insect fossils trapped in amber [14, 15]; therefore, their systematic placement must be inferred from their morphological characters. In Zoraptera, the most important characters are those involving the male genitalia, but these are usually enclosed in the abdomen and only rarely visible [16].

Altogether, 12 fossil species of Zoraptera are currently recognized from the Mesozoic [1]. One species is reported from the lower Cretaceous Jordanian amber (Albian) [17], and 11 species are known from the upper Cretaceous amber (Cenomanian) of northern Myanmar [1]. Fossil zorapterans are classified partly in *Zorotypus *sensu stricto, partly in the monotypic genus *Xenozorotypus* Engel & Grimaldi, 2002, and partly in the exclusively fossil subgenus *Octozoros* Engel, 2003, which was established for species with eight antennomeres [6, 18]. All of these taxa are included in a single family, Zorotypidae [9, 13].

In this study, we compared the observable morphological characteristics of all known Mesozoic Zoraptera. On this basis, we propose their classification and the diagnostic characteristics of individual taxonomic groups. Our conclusions are based partly on the available literature and images of Mesozoic species and partly on a new set of fossils in Burmese amber in which the diagnostically significant parts of the genitalia and other characters are observable. Based on detailed examinations and comparisons of these characters we were able to classify most Mesozoic Zoraptera species into the currently accepted system, which was created based on extant taxa. To evaluate the phylogenetic relationships of newly established fossil Zoraptera taxa, we focused on estimating the evolutionary histories of selected morphological features through ancestral character state reconstruction (ASR).

## Results

### Evolution of character states

**Fig. 1:**
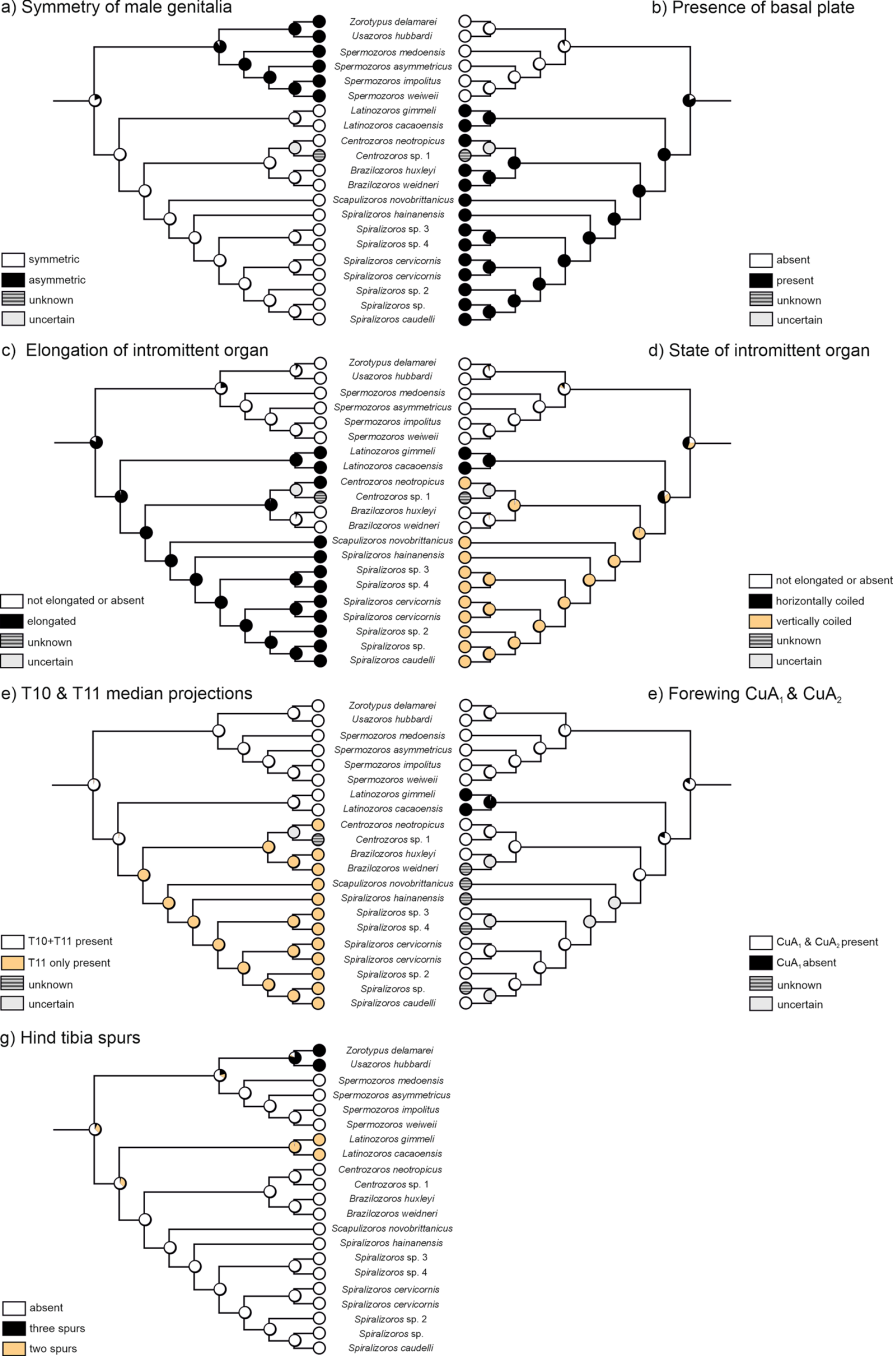
Ancestral state reconstruction assessed by maximum likelihood criterion for selected morphological characters of Zoraptera

**Fig. 2:**
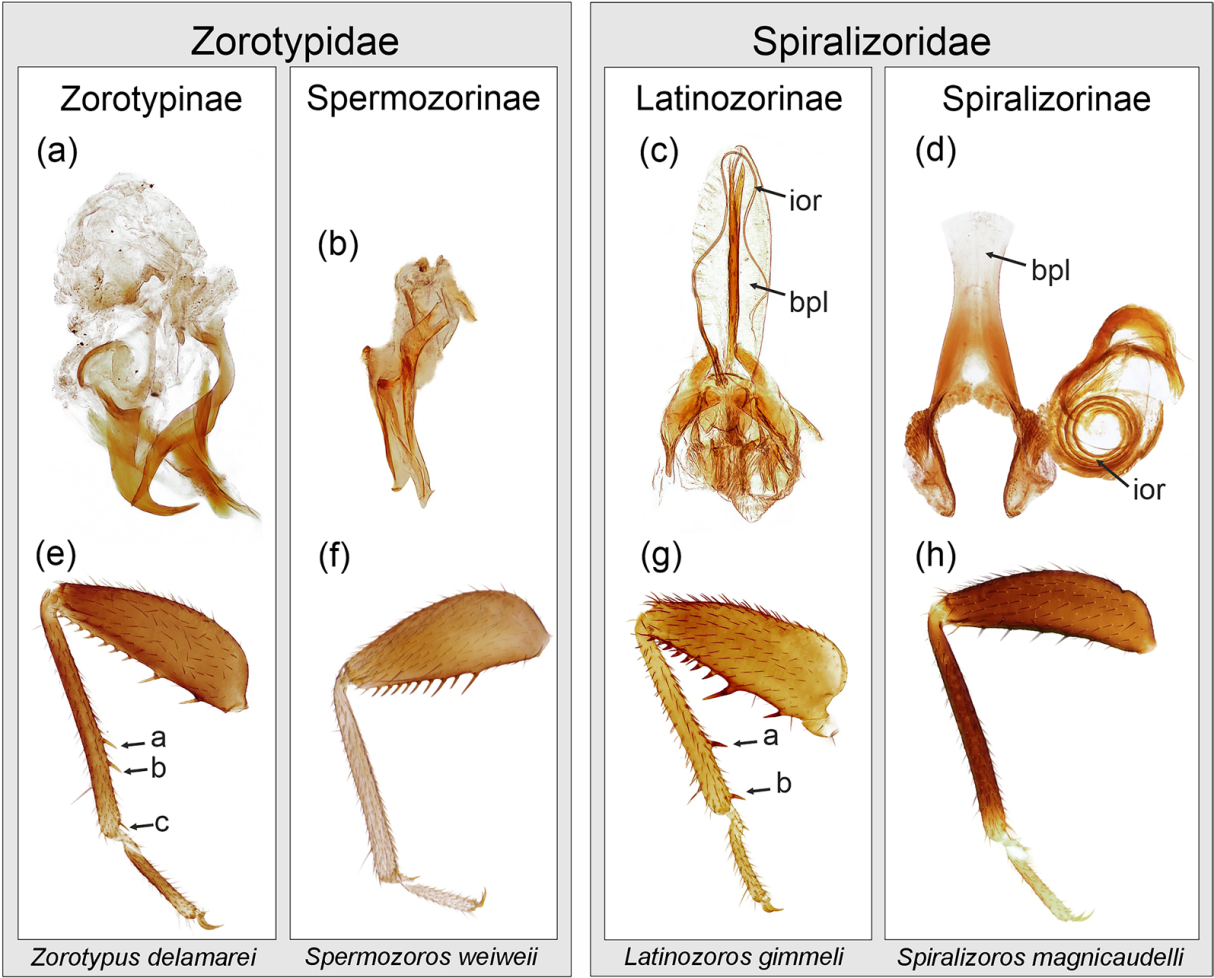
Comparison of diagnostic characteristics for currently recognized Zoraptera subfamilies. **a**–**d** Male genitalia, dorsal view. **e**–**h** Hindleg, lateral view. a–c, metatibial spurs; bpl, basal plate; ior, intromittent organ. Comment: Genital (d) is moved from its original position, which is perpendicular to the basal plate

Based on a study of representatives of all recent Zoraptera genera, three types of male copulatory organs have been identified. These types are observable in fossil representatives and can be used for taxonomic classification at the genus level. With respect to the symmetry of the male copulatory organs, we recognize the following states: symmetric vs. asymmetric, absence vs. presence of a basal plate, elongated vs. not elongated or absent intromittent organ, horizontally coiled vs. vertically coiled intromittent organ (Figs. 1 and 2). According to our estimates, the ancestral state of Zoraptera was probably a symmetric configuration of the genitalia (Fig. 1a), whereas the asymmetric state is probably a synapomorphy of Zorotypidae. The ancestral states of both, the basal plate and the elongated intromittent organ, were probably the presence in the common ancestor of Zoraptera (Fig. 1b, c). In contrast, the absence of a basal plate and not elongated or missing intromittent organ are probably apomorphic characteristics of Zorotypidae. Within Spiralizoridae, three states occur: (a) a horizontally coiled intromittent organ in Latinozorinae; (b) a vertically coiled intromittent organ in the majority of Spiralizorinae; and (c) an intromittent organ not elongated in *Brazilozoros* Kukalova-Peck & Peck, 1993 and *Aspiralizoros* Kočárek & Kočárková, 2024. We consider the absence of elongated intromittent organs in *Brazilozoros* and *Aspiralizoros* to be a secondary reduction, which can be considered as homoplasy. However, the ancestral state of the elongation type of the intromittent organ in the common ancestor of Spiralizoridae cannot be assessed with certainty. According to the ASR (Fig. 1d), the most probable scenario is that the horizontally coiled intromittent organ is the ancestral state and the vertically coiled intromittent organ is the derived state. Therefore, we suppose the vertically coiled intromittent organ to be a synapomorphy of the Spiralizorinae subfamily. In addition to the male genitalia, we examined the state of projection on abdominal tergites T10 and T11. We recorded the following states: (a) presence on T10 + T11; and (b) presence on T11 only. According to our estimates (Fig. 1e), the ancestral state of Zoraptera was probably the presence of protuberances on both T10 and T11, whereas the reduction in the protuberance on T10 seems to be an apomorphy of Spiralizorinae.

Kočárek et al. [13] identified three stages of development of metatibial spurs: (a) three spurs in Zorotypinae (an additional spur may also be developed; see Matsumura et al. [4]), (b) two spurs in Latinozorinae, and (c) an absence of spurs in Spermozorinae and Spiralizorinae (Fig. 2). According to our estimates, the ancestral state was probably the absence of metatibial spurs, whereas the presence of spurs is a derived state (Fig. 1g). We consider the presence of three metatibial spurs to be a synapomorphy of the subfamily Zorotypinae and the presence of two spurs to be a synapomorphy of Latinozorinae (Fig. 1g). As the development of metatibial spurs correlates with the male copulatory organs character, as reported in previous studies of recent representatives [13], this feature can be used for the classification of fossils where information on copulatory organs is unavailable.

**Fig. 3:**
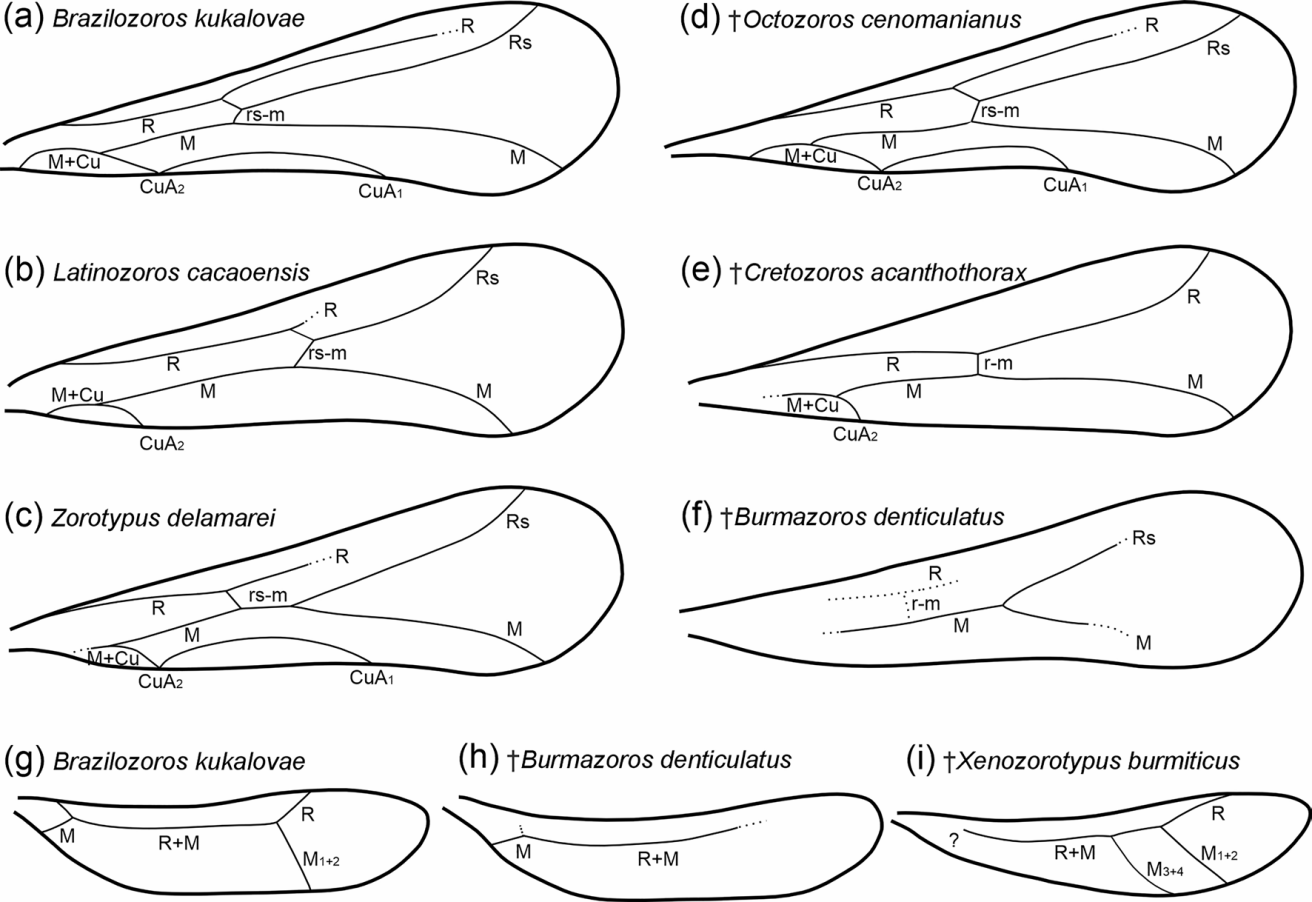
Schematic drawings of wing venation patterns of extant and fossil Zoraptera. (a-f) Forewings. **g**–**i** Hindwings. **a**–**c**, **g** Extant Zoraptera species. **d**–**f**, **h**–**i** Mesozoic Zoraptera species. Abbreviations: Cu, cubitus vein; CuA, anterior cubitus vein; M, media vein; R (Rs), radius vein (radial sector), r(rs)-m, crossvein between R(Rs) and M

A comparison of all recent Zoraptera species with wing venation patterns described to date revealed a high degree of uniformity in the shape of individual veins. Two states concerning CuA on the forewings of recent representatives were recorded: (a) the presence of CuA_1_ + CuA_2_ (Fig. 3a, c), and b) the absence of CuA_1_ (Fig. 3b). According to our estimates, the ancestral state was probably the presence of CuA_1_ + CuA_2_ (Fig. 1e), whereas the absence of CuA_1_ is a derived state that is apomorphic for *Latinozoros* Kukalova-Peck & Peck, 1993 (Fig. 1e). The development of veins on the hindwings has been uniform in recent species (Fig. 3g). In fossil species, both wings with fully developed venation are considered plesiomorphic, and derived states are associated with venation reduction (Fig. 3e, f). In addition to the reduction of CuA_1_ on the forewings, which was recorded in recent representatives, the absence of the Rs vein and the absence of CuA_1_ + CuA_2_, accompanied by a partial reduction of other veins (R, r-m, m), was recorded (Fig. 3e, f). We consider the absence of CuA_1_ and the absence of CuA_1_ + CuA_2_ and Rs (associated with the reduction of other veins) as homoplasies. Differences in the development of the M veins on the forewings were observed only in fossil species, with all recent species having M_1+2_ veins (Fig. 3g). We consider the absence of M_1+2_ veins, as well as their presence together with M_3+4_ veins, to be synapomorphies (Fig. 3h, i).

### Phylogeny and systematic placement of Mesozoic Zoraptera

**Fig. 4:**
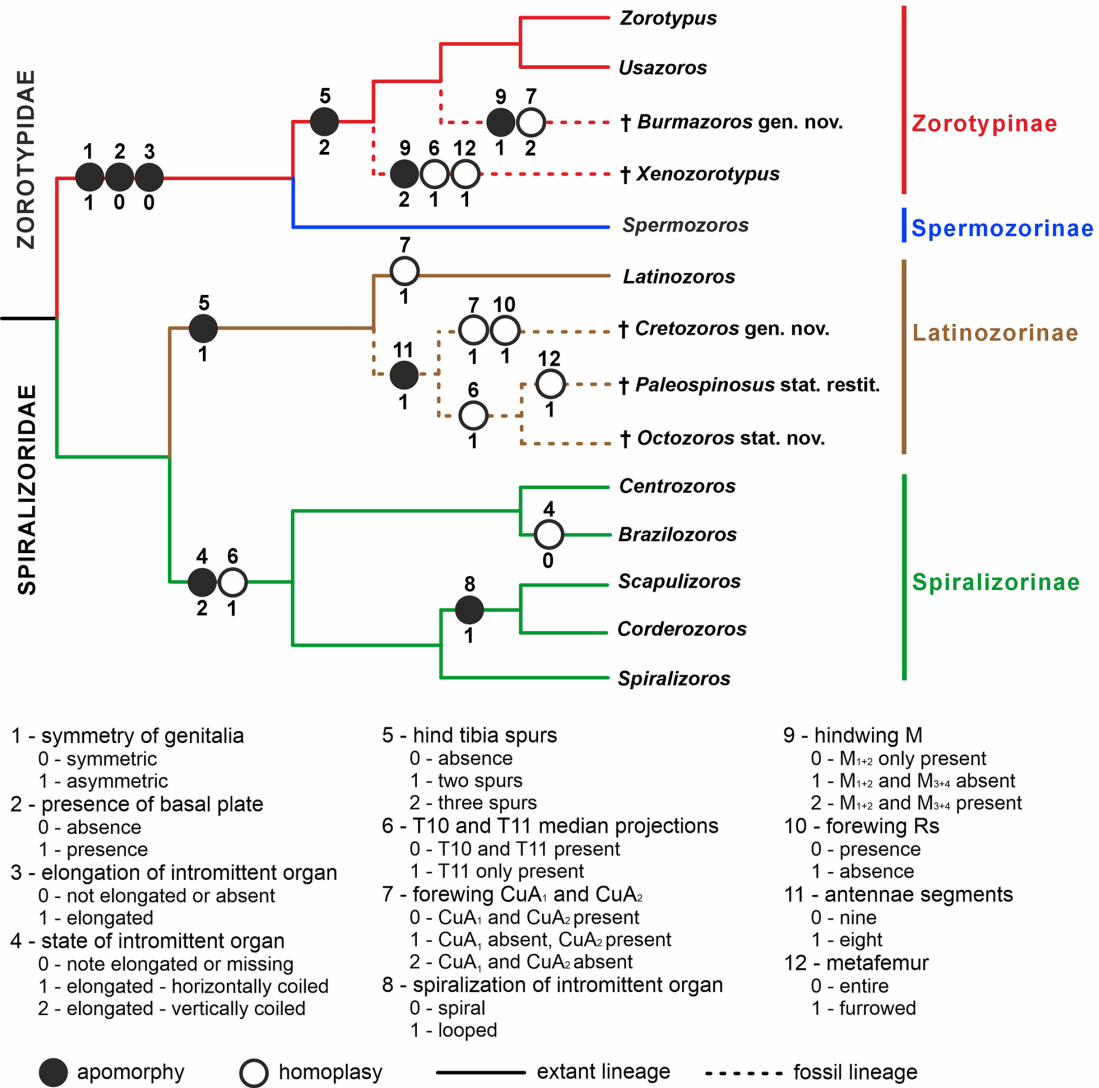
Simplified phylogenetic scheme of Zoraptera evolution with probable placement of the fossil genera *Cretozoros* gen. nov., *Burmazoros* gen. nov., *Octozoros* Engel, 2003, stat. nov., *Paleospinosus* Kaddumi, 2005, stat. restit., and *Xenozorotypus* Engel & Grimaldi, 2002. The phylogenetic scheme is modified from that of Kočárek et al. [13]. The solid lines depict the phylogenetic relationships based on molecular phylogeny; the dashed lines depict the probable phylogenetic relationships of the fossil taxa based on apomorphies and homoplasies. The numbers (1–12) above the circles refer to character numbers, and the numbers below the circles (0–2) refer to character states. States of characters 1–7 are polarized by ancestral state character reconstruction (ASR); states of additional characters 8–12 were not evaluated by ASR (see the text for details)

Morphological characteristics were used to propose a classification of Mesozoic Zoraptera and to evaluate their phylogenetic relationships (Fig. 4). The evolution of the morphological characters was estimated using ancestral state reconstruction (see Fig. 1). Both Zorotypidae and Spiralizoridae have well-defined copulatory organ characters, with mutually correlated states of symmetry (1), the presence of a basal plate (2) and the elongation and the state of the intromittent organ (3 and 4) (Fig. 4). The only exception is the secondary reduction of the intromittent organ in *Brazilozoros* and *Aspiralizoros* (Spiralizoridae: Spiralizorinae). Probable ancestral states were the symmetry of genitalia and the presence of a basal plate and an elongated intromittent organ (Figs. 1, 2). The combination of these ancestral morphological characteristics of the copulatory organs defines the family Spiralizoridae, while derived apomorphic characteristics define the family Zorotypidae (Fig. 4). The presence of MPs on abdominal tergites T10 and T11 in males was identified as the ancestral state, whereas the reduction of the projection on T10 was identified as the derived state defining Spiralizoridae: Spiralizorinae (Figs. 1e, 4). The absence of spurs on the metatibiae was evaluated as an ancestral condition. Derived states define the subfamily Zorotypinae (Zorotypidae) having three spurs, and the subfamily Latinozorinae (Spiralizoridae) having two spurs (Figs. 2 and 4). In Zoraptera, the ancestral state of the forewings was assessed as having fully developed veins. The derived state has reduced veins, which have appeared independently (homoplasy) in the genera *Latinozoros*, *Cretozoros* gen. nov., and *Burmazoros* gen. nov. (Fig. 4).

Based on the synapomorphic morphological characters for which ASR analysis was performed, the fossil genera *Cretozoros* gen. nov., *Burmazoros* gen. nov., *Paleospinosus* Kaddumi, 2005, stat. restit., *Octozoros* Engel, 2003, stat. nov. and *Xenozorotypus*, can be classified into a system of families and subfamilies. We identified some morphological character states in the fossil genera that are not present in recent representatives and therefore could not be directly polarized (Tables 1 and 2). In addition to the presence of fully developed veins on the forewings (*Octozoros* Engel, 2003, stat. nov. and *Xenozorotypus*), the following states were identified: reduced CuA_1_ and simultaneously not developed Rs (*Cretozoros* gen. nov.); and reduction of both CuA_1_ + CuA_2_, with partial reduction of R, M and Rs (*Burmazoros* gen. nov., probably also *Paleospinosus* Kaddumi, 2005, stat. restit.). Given the identified ancestral state, we consider the reduction of CuA_1_ to be an homoplastic apomorphy of the genera *Latinozoros* and *Cretozoros* gen. nov., and the reduction of both CuA_1_ + CuA_2_, with partial reduction of R, M and Rs, to be apomorphy of *Burmazoros* gen. nov. (Fig. 4). The ancestral state of the hindwing venation character is the presence of M_1+2_, which occurs in all recent Zoraptera species with known wings, as well as in the fossil genera *Cretozoros* gen. nov., *Paleospinosus* Kaddumi, 2005, stat. restit., *Octozoros* Engel, 2003, stat. nov., and *Xenozorotypus* (Fig. 3). The absence of M_1+2_ was observed in the genus *Burmazoros* gen. nov., and we consider it to be an apomorphy of this genus (Fig. 4 and Table 2). Conversely, the presence of M_3+4_ was found only in the genus *Xenozorotypus*, for which we consider it to be an apomorphy (Fig. 4, Table 2).

**Table 1:** Genitalic characters and their states in Mesozoic genera of Zoraptera. Apomorphic character states at family, subfamily or genus level are in bold

Mesozoic genus	Systematic placement	Genitalia symmetry	Basal plate	Intromitt. organ	State of intromitt. organ	Rod-shaped sclerites	T10/T11 projections	Ctenidia on T10
†*Burmazoros* gen. nov.	Zorotypidae/Zorotypinae	**Asymmetrical**	**Absent**	**Absent**	Not elongated / absent	Present	T10 + T11	Absent
†*Xenozorotypus* Engel & Grimaldi, 2002	Zorotypidae/Zorotypinae	N/A	N/A	N/A	N/A	N/A	**T11**	Present
†*Octozoros* Engel, 2003, stat. nov.	Spiralizoridae/Latinozorinae	Symmetrical	Present	Present	**horizontally coiled**	Absent	**T11**	Present
†*Cretozoros* gen. nov.	Spiralizoridae/Latinozorinae	Symmetrical	Present	Present	**horizontally coiled**	Present	T10 + T11	Absent
†*Paleospinosus* Kaddumi, 2005, stat. rest.	Spiralizoridae/Latinozorinae	N/A	N/A	N/A	N/A	N/A	**T11**	Absent

**Table 2:** Nongenitalic characters and their states in Mesozoic genera of Zoraptera. Apomorphic character states at family, subfamily or genus level are in bold

Mesozoic genus	Systematic placement	Forewing CuA	Forewing Rs	Hindwing M_3_ + M_4_	Hindwing M_1_ + M_2_	Metatibia spurs	Antennomeres	Metafemur furrowing
†*Burmazoros* gen. nov.	Zorotypidae/Zorotypinae	**Absent**	Present	Absent	**Absent**	3	9	No
†*Xenozorotypus* Engel & Grimaldi, 2002	Zorotypidae/Zorotypinae	CuA_1_ + CuA_2_	Present	**Present**	Present	3	9	**Yes**
†*Octozoros* Engel, 2003, stat. nov.	Spiralizoridae/Latinozorinae	CuA_1_ + CuA_2_	Present	Absent	Present	**2**	**8**	No
†*Cretozoros* gen. nov.	Spiralizoridae/Latinozorinae	**CuA**_**2**_	**Absent**	Absent	Present	**2**	**8**	No
†*Paleospinosus* Kaddumi, 2005, stat. rest.	Spiralizoridae/Latinozorinae	**(?) Absent**	**(?) Absent**	Absent	Present	**2**	**8**	**Yes**

With respect to the development of MPs on the abdominal tergites T10 and T11 in males, fossil specimens exhibit both the ancestral state (T10 + T11, as seen in *Burmazoros* gen. nov. and *Cretozoros* gen. nov.) and the derived state (T11 only, as seen in *Paleospinosus* Kaddumi, 2005, stat. restit., *Octozoros* Engel, 2003, stat. nov., and *Xenozorotypus*) (Table 1). The latter state was employed in generic diagnoses. The reduction of the projection on T10 appears to have occurred repeatedly during the evolution of the group and is considered homoplasy. For genus-level diagnoses, the nonpolarized character of furrowed hind tibiae (*Xenozorotypus, Paleospinosus*) and the derived shape of the male abdominal sternum S8 (*Burmazoros* gen. nov. and *Paleospinosus* Kaddumi, 2005, stat. restit.) were also used (Fig. 4 and Table 2).

In fossil representatives of Zoraptera, the number of antennomeres has decreased from nine (which we consider be the ancestral state of this character) to eight in *Cretozoros* gen. nov., *Paleospinosus* Kaddumi, 2005, stat. restit., and *Octozoros* Engel, 2003, stat. nov. We define a clade comprising *Cretozoros* gen. nov., *Paleospinosus* Kaddumi, 2005, stat. restit. and *Octozoros* Engel, 2003 (Fig. 4; Tables 3 and 4) by an apomorphic state of the antennae (eight segments). We consider this clade to be the sister group of *Latinozoros*, which has an ancestral state of this character (nine segments).

**Table 3:** An updated higher-level classification of Zoraptera

**Zorotypidae** Silvestri, 1913	
** Zorotypinae** Silvestri, 1913	
* Zorotypus* Silvestri, 1913	
* Usazoros* Kukalova-Peck & Peck, 1993	
†*Burmazoros* gen. nov	
†*Xenozorotypus* Engel & Grimaldi, 2002	
** Spermozorinae** Kočárek, Horká & Kundrata, 2020	
* Spermozoros* Kočárek, Horká & Kundrata, 2020	
**Spiralizoridae** Kočárek, Horká & Kundrata, 2020	
** Latinozorinae** Kočárek, Horká & Kundrata, 2020	
* Latinozoros* Kukalova-Peck & Peck, 1993	
†*Octozoros* Engel, 2003, stat. nov	
†*Cretozoros* gen. nov	
†*Paleospinosus* Kaddumi, 2005, stat. restit	
** Spiralizorinae** Kočárek, Horká & Kundrata, 2020	
* Aspiralizoros* Kočárek & Kočárková, 2024	
* Spiralizoros* Kočárek, Horká & Kundrata, 2020	
* Centrozoros* Kukalova-Peck & Peck, 1993	
* Brazilozoros* Kukalova-Peck & Peck, 1993	
* Scapulizoros* Kočárek, Horká & Kundrata, 2020	
* Cordezoros* Kočárek, Horká & Kundrata, 2020

**Table 4:** The checklist of Mesozoic Zoraptera

**Zorotypidae** Silvestri, 1913
** Zorotypinae** Silvestri, 1913
†*Burmazoros* gen. nov
†*B. denticulatus* (Yin, Cai & Huang, 2018), comb. nov
(= †*Zorotypus oligophleps* Liu, Zhang, Cai & Li, 2018)
†*Xenozorotypus* Engel & Grimaldi, 2002
†*X. burmiticus* Engel & Grimaldi, 2002
**Spiralizoridae** Kočárek, Horká & Kundrata, 2020
** Latinozorinae** Kočárek, Horká & Kundrata, 2020
†*Octozoros* Engel, 2003
†*O. nascimbenei* (Engel & Grimaldi, 2002), comb. nov
†*O. robustus* (Liu, Zhang, Cai & Li, 2018), stat. restit., comb. nov
(= †*Zorotypus hirsutus* Mashimo, 2018, syn. nov.)
†*O. cenomanianus* (Yin, Cai & Huang, 2018), comb. nov
†*O. pecten* (Mashimo, 2019), comb. nov
†*Cretozoros* gen. nov
†*C. acanthothorax* (Engel & Grimaldi, 2002), comb. nov
(= †*Z. hukawngi* Chen & Su 2019, syn. nov.)
†*C. pusillus* (Chen & Su, 2019), comb. nov
†*Paleospinosus* Kaddumi, 2005, stat. restit
†*Paleospinosus hudae* Kaddumi, 2005
** Incertae sedis in Zoraptera:**
†*Zorotypus cretatus* Engel & Grimaldi, 2002
†*Zorotypus dilaticeps* Yin, Cai, Huang & Engel, 2018

The fossil genera *Burmazoros* gen. nov. and *Xenozorotypus*, were classified in Zorotypidae: Zorotypinae based on a combination of synapomorphic characteristics (Fig. 4). We propose *Xenozorotypus* as a sister to the clade containing *Zorotypus*, *Usazoros* Kukalova-Peck & Peck, 1993, and *Burmazoros*, based on the apomorphic states of M_1+2_ and M_3+4_ presence on the hindwings, and the absence of CuA_1_ + CuA_2_ on the forewings. *Burmazoros* gen. nov. we propose as sister to the clade comprising recent *Zorotypus* and *Usazoros*, which share ancestral wing venation states. *Burmazoros* gen. nov. is defined by apomorphic absence of M_1+2_. The genera *Cretozoros* gen. nov., *Paleospinosus* Kaddumi, 2005, stat. restit. and *Octozoros* Engel, 2003, stat. nov. were classified in Spiralizoridae: Latinozorinae based on synapomorphic state of metatibia spurs (Fig. 4). The genera *Paleospinosus* and *Octozoros* share a median projection on T11 (homoplasy) and we suppose their sister relationships (Fig. 4). Clade *Paleospinosus*/*Octozoros* we suppose sister to *Cretozoros* defined by absence of CuA_1_ and Rs on the forewings (see Fig. 4 and Table 2).

Two Mesozoic species, *Zorotypus cretatus* Engel & Grimaldi, 2002 and *Zorotypus dilaticeps* Yin, Cai, Huang & Engel, 2018, could not be classified within the Zoraptera system because of poor fossil preservation, the availability of only apterous specimens, and the lack of information on male genitalia.

### Systematic paleontology

**Order Zoraptera Silvestri, 1913**

**Family Zorotypidae Silvestri, 1913**

**Subfamily Zorotypinae Silvestri, 1913**

**Genus *Burmazoros*** **gen. nov.**

urn:lsid:zoobank.org:act:089A6025-92CB-4242-97CE-C0FFB7980D85.

**Type species** *Zorotypus denticulatus* Yin, Cai & Huang, 2018, here designated.

**Etymology** The generic name refers to the occurrence of this taxon in Burmese amber, in combination with a suffix derived from the word base of the order name. Gender masculine.

**Diagnosis** *Burmazoros* gen. nov. is distinguished by the following unique combination of characters: antennae 9-segmented; metafemur with a middle spine larger than the basal spine; metatibia with three acute spines at midlength and at the preapical and apical portions; and ctenidia absent on T10, with T10 and T11 each bearing a small conical median projection (MP) slightly curved upward. Male genitalia probably asymmetrical (cf. Fig. 2 in [19]), with rod-shaped accessory sclerites developed laterally, and without a basal plate and elongated intromittent organ. *Burmazoros* gen. nov. differs from the recent *Zorotypus* Silvestri, 1913, in its reduced wing venation: CuA absent in forewings; hindwings with M_1+2_ absent, and short M fused with R in the middle region and not reaching the wing margin (Fig. 3h).

**Systematic placement** *Burmazoros* gen. nov. is herein assigned to Zorotypidae: Zorotypinae based on the following characters: asymmetrical male genitalia, absence of a basal plate and an elongated intromittent organ, and the presence of three metatibial spurs (Tables 1 and 2). We assume that this genus is related to the recent genera *Zorotypus* and *Usazoros*s.

**Species included** *Burmazoros denticulatus* (Yin, Cai & Huang, 2018) (= *Zorotypus oligophleps* Liu, Zhang, Cai & Li, 2018).

**Distribution** Myanmar, Kachin State, Myitkyina District, Hukawng Valley, Burmese amber (Upper Cretaceous, lower Cenomanian).

***Burmazoros denticulatus*** **(Yin, Cai & Huang, 2018), comb. nov.**

*Zorotypus denticulatus* Yin, Cai & Huang, 2018: 169 [19].

 = *Zorotypus oligophleps* Liu, Zhang, Cai & Li, 2018: 260 [20].

**Comments** This species is known only from the type specimen, which is a well-preserved alate male [19]. The metafemur has a distinct arrangement of spines; the metatibia bears three strong spurs on the distal third. Male genitalia are partly visible, and asymmetrical, rod-shaped accessory sclerites are developed laterally. Short MPs are developed on T10 and T11.

Yin et al. [21] synonymized *Zorotypus oligophleps* Liu, Zhang, Cai & Li, 2018, with *Zorotypus denticulatus* Yin, Cai & Huang, 2018. These species share similar head and pronotum forms, identical configurations and proportions of antennomeres and similarly reduced wing venations [19]. The only difference is in the number of metatibial spurs. *Burmazoros denticulatus* has three spurs, and Z. *oligophleps* has two spurs [21]. Although the number and arrangement of the metatibial spurs are important diagnostic characters, the absence of the middle spur in the holotype of *Z. oligophleps* may actually represent breakage rather than a true absence. Until the number of spurs is confirmed on newly found individuals of this species, we provisionally retain Z. *oligophleps* in synonymy.

**Genus** ***Xenozorotypus*** **Engel & Grimaldi, 2002**

*Xenozorotypus* Engel & Grimaldi, 2002: 12 [22].

**Type species** *Xenozorotypus burmiticus* Engel & Grimaldi, 2002, by original designation.

**Systematic placement** *Xenozorotypus* is herein assigned to Zorotypidae: Zorotypinae. It shares three metatibial spurs with members of Zorotypinae (Table 1), but the male genitalia are not observable in the only known specimen of this species [22]. A characteristic apomorphy is the venation of the hindwing, in which M_3+4_ is present (Table 2). We hypothesize that this genus is related to the recent genera *Zorotypus* and *Usazoros,* and the fossil genus *Burmazoros* gen. nov.

**Species included** *Xenozorotypus burmiticus* Engel & Grimaldi, 2002.

**Distribution** Myanmar, Kachin State, Myitkyina District, Hukawng Valley, Burmese amber (Upper Cretaceous, lower Cenomanian).

***Xenozorotypus burmiticus*** **Engel & Grimaldi, 2002**

*Xenozorotypus burmiticus* Engel & Grimaldi, 2002: 12 [22].

**Comments** This species is known only from the holotype, which is a rather poorly preserved male specimen [22]. The metafemur shows an exceptionally deep ventral furrow extending from the apex to the midpoint; the metatibia bears three strong spurs regularly spaced in the distal two-thirds, and a basal spine is present on the ventral surface of the metatibia. Procurved MP is present and clearly visible, but it is unclear if it is a projection of the T10 or T11 sclerite. According to its shape and position, we suppose it is a T11 derivative. Hindwing venation with M_3+4_ is present and characteristic (Fig. 3i). The genitalia are not observable.

**Family Spiralizoridae Kočárek, Horká & Kundrata, 2020**

**Subfamily Latinozorinae Kočárek, Horká & Kundrata, 2020**

**Genus** ***Octozoros*** **Engel, 2003, stat. nov.**

*Octozoros* Engel, 2003: 148 (as subgenus of *Zorotypus*).

(Figs. 5, 6).

**Fig. 5:**
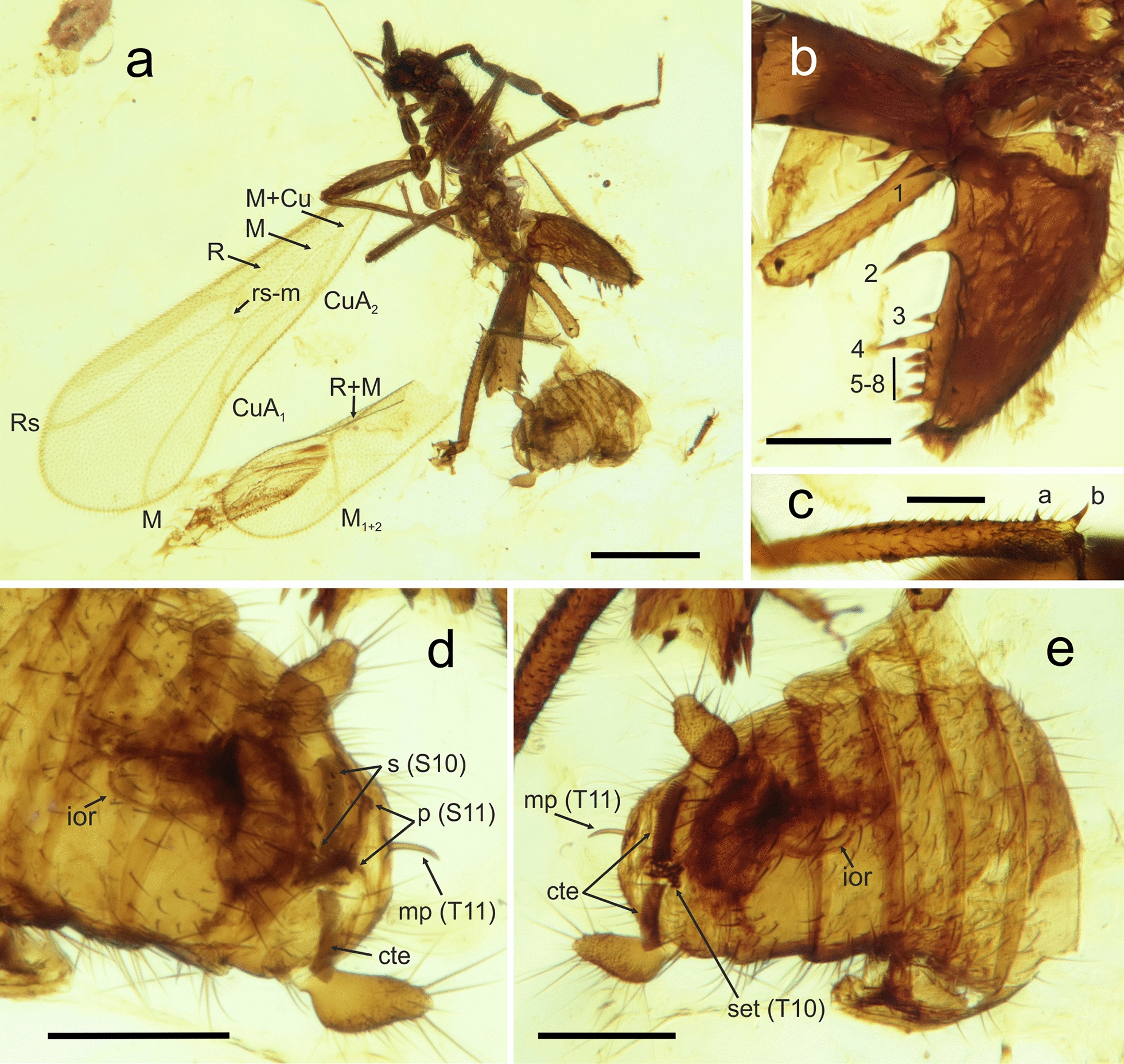
Habitus and details of *Octozoros cenomanianus* (Yin, Cai & Huang, 2018), comb. nov., specimen PK344Bu, male. **a** General habitus. **b** Detail of the left metafemur, lateral view. **c** Detail of the right metatibia, lateral view. **d** Abdomen, ventral view. **e** Abdomen, dorsal view. a, b, metatibial spurs; cte, ctenidium; Cu, cubitus vein; CuA, anterior cubitus vein; ior, intromittent organ; M, media vein; R (Rs), radius vein; mp, median upcurved projection; p, projections; s, spines; set, thickened setae; S10–S11, sternites; T10–T11, tergites; 1–8, metafemoral spurs. Scale bars: 0.5 mm (**a**), 0.2 mm (**b**, **d**, **e**), and 0.1 mm (**c**)

**Fig. 6:**
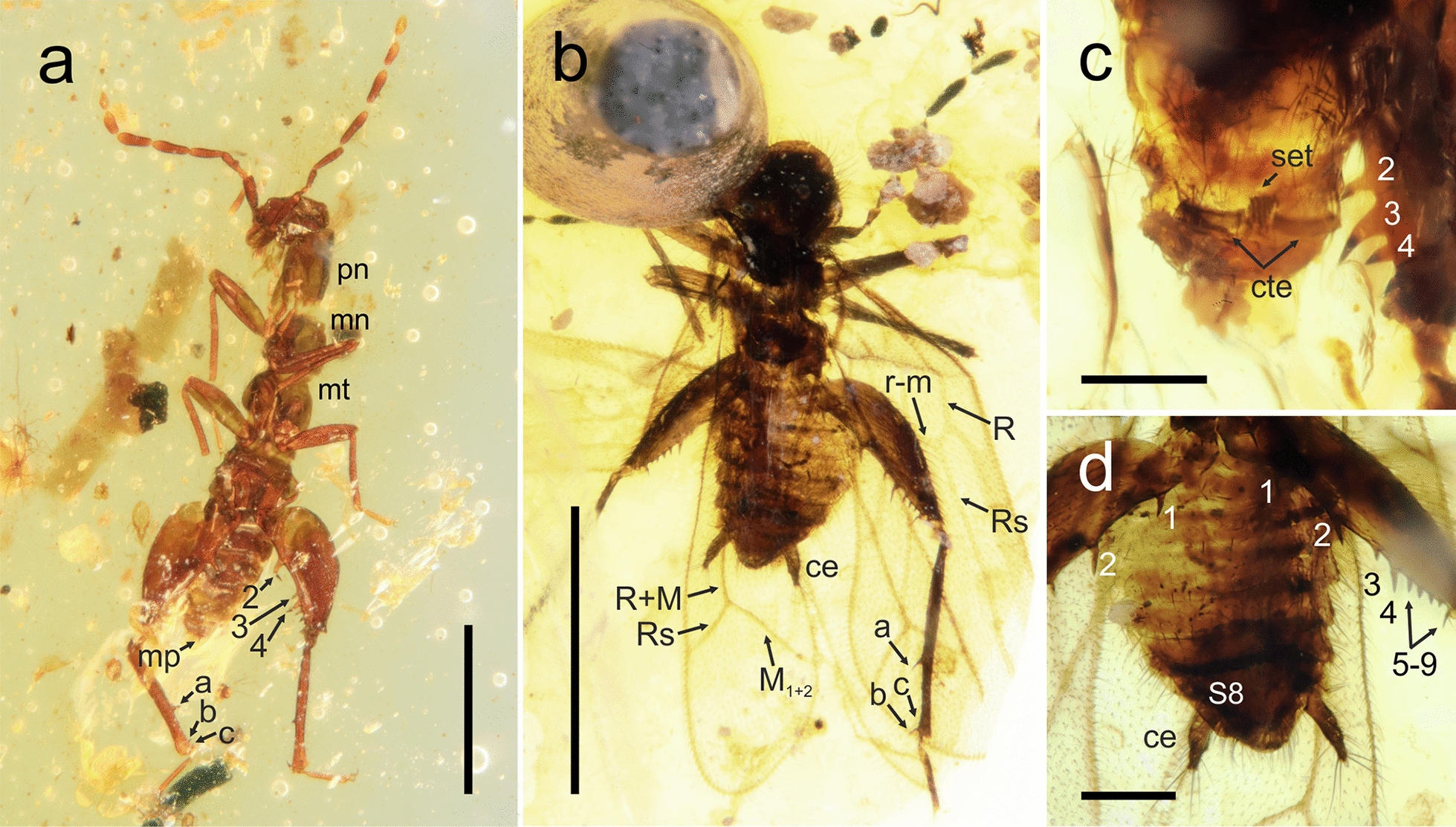
Habitus and details of *Octozoros robustus* (Liu, Zhang, Cai & Li, 2018), stat. restit., comb. nov. **a**, **c** Specimen PK343Bu, male; **b**, **d** specimen PK348Bu, female. **a** General habitus of an apterous male, ventral view. **b** General habitus of a winged female, ventral view. **c** Detail of the male abdomen, dorsal view. **d** Detail of the female abdomen, ventral view. a, b, c, metatibial spurs; cte, ctenidium; M, media vein; mn, mesonotum; mp, median upcurved projection; mt, metanotum; pn, pronotum; R (Rs), radius vein; set, tickened setae; S8, sternite 8; 1–9, metafemoral spurs. Scale bars: 1.0 mm (**a**, **b**) and 0.2 mm (**c**, **d**)

**Type species** *Zorotypus nascimbenei* Engel & Grimaldi, 2002, by original designation (in subgenus *Octozoros* Engel, 2003).

**Updated diagnosis** *Octozoros* is characterized by the following unique combination of characters: antennae 8-segmented; forewings with fully developed venation; R divided into R and Rs in the midpart; Rs continuing from the radial stem and connected to M by a short Rs-M crossvein; M and Rs reaching the posterior wing margin near the wing apex; CuA_1_ and CuA_2_ present (Fig. 3d); ventral margin of the metafemur not furrowed or has only a shallow furrow; hind tibiae with two robust spurs, one in the distal third to fifth and the second distally; in some species, e.g., *O. robustus* (Liu, Zhang, Cai & Li, 2018) and *O. pecten* (Mashimo, 2019), another tiny apical spine developed on the inside of the ventral surface; in males, T10 with two rows of thick setae arranged as a comb (ctenidium) on both sides; the central region of T11 has long and thin upcurved MPs, which are absent on T10; and male genitalia symmetrical with a robust basal part and a tongue-like anterior process encircled by the elongated intromittent organ, with the absence of lateral rod-shaped accessory sclerites.

Within Latinozorinae, *Octozoros* differs from *Burmazoros* gen. nov. in the presence of CuA_1_ on the forewings, ctenidia on T10, and only T11 with upcurved MP. *Octozoros* differs from the recent *Latinozoros* Kukalova-Peck & Peck, 1993 in terms of the number of MPs in the male abdomen (*Octozoros* has MP only on T11, whereas *Latinozoros* has MPs on both T10 and T11) and in the number of antennomeres (antennae of *Octozoros* are composed of 8 antennomeres in adults of both sexes, whereas the antennae of *Latinozoros* are always composed of 9 antennomeres in adults).

**Systematic placement** Subgenus *Octozoros* (in *Zorotypus*) is herein elevated to the genus level and assigned to Spiralizoridae: Latinozorinae. *Octozoros* shares with *Latinozoros* a similar pattern of symmetrical male genitalia with the basal plate encircled by the elongated intromittent organ (Tables 1 and 2). *Octozoros* Engel, 2003, stat. nov. shares an apomorphic state of the antennae (eight segments) with *Cretozoros* gen. nov. and *Paleospinosus* Kaddumi, 2005, stat. restit. and we consider *Octozoros/Cretozoros/Paleospinosus* clade to be the sister group of *Latinozoros*, which has an ancestral state of this character (nine segments).

**Species included** *Octozoros cenomanianus* (Yin, Cai & Huang, 2018); *O*. *nascimbenei* (Engel & Grimaldi, 2002); *O. pecten* (Mashimo, 2019); *O. robustus* (Liu, Zhang, Cai & Li, 2018), stat. restit. (= *Z. hirsutus* Mashimo, 2018, syn. nov.).

**Distribution** Myanmar, Kachin State, Myitkyina District, Hukawng Valley, Burmese amber (Upper Cretaceous, lower Cenomanian).

***Octozoros cenomanianus*** **(Yin, Cai & Huang, 2018), comb. nov.**

*Zorotypus cenomanianus* Yin, Cai & Huang, 2018: 169 [19].

(Fig. 5).

**Materials examined** One adult specimen: alate male, PK344Bu (Burmese amber).

**Supplementary description** Based on the study of new material, we supplement the description of *C. cenomanianus* with the following morphological characters, which are not observable in the holotype [19]. Both specimens known thus far are adult males; immature stages and female specimens remain undescribed.

**Legs** Metatibia with two stout spines, one in the distal 1/5th, second distally (Fig. 5a–c). Spines not aligned on the axis of the tibia; proximal spine located on the outer edge and distal spine located on the inner edge of the tibia (Fig. 5c). Spines thus oriented obliquely to each other (Fig. 5b). Meta-pretarsus with pair of thin bristles; empodium reduced to short hair-like structure.

**Male genitalia** Symmetrical, composed of a robust basal part without recognizable details and a proximally oriented protrusion (basal plate) horizontally encircled by the elongated intromittent organ. Intromittent organ spirally wrapped around the basal plate with two convolutions.

***Octozoros nascimbenei*** **(Engel & Grimaldi, 2002), comb. nov.**

*Zorotypus nascimbenei* Engel & Grimaldi, 2002: 7 [22].

**Comments** This species is known only from the type specimen, which is an alate female [22]. The pattern of metafemur supination is species-specific, with spines 1 and 2 robust and the remaining spines short, reaching maximally half of the length of spines 1 and 2. The metatibia bears two strong spurs in the distal third. The pronotum has a shallow depression on the anterior margin. Males have not been recorded; therefore, the male genitalia characters, arrangement of ctenidia on T10, and MPs on T10 and T11 (presence and shape) cannot be evaluated. The species is assigned in *Octozoros* stat. nov. by the combination of the following observable characters: antennae 8-segmented, CuA_1_ present, and hind tibiae with two spurs in the distal third.

***Octozoros robustus*** **(Liu, Zhang, Cai & Li, 2018), stat. restit., comb. nov.**

*Zorotypus robustus* Liu, Zhang, Cai & Li, 2018: 260 [20].

 = *Zorotypus hirsutus* Mashimo, 2018: 563 [23], syn. nov.

(Fig. 6).

**Materials examined** Four adult specimens: two alate females, PK348Bu and PK164Bu; one apterous male, PK343Bu; and one apterous female, PK336Bu (all Burmese amber).

**Taxonomic remarks** Yin et al. [21] synonymized *Zorotypus robustus* with *Zorotypus cenomanianus* (currently in the genus *Octozoros*). Based on the comparison of original descriptions as well as on the study of new material of both species (see Materials and Methods), these two species are recognized as distinctive. Therefore, *Z. robustus* is resurrected from synonymy and considered a valid species in the genus *Octozoros*. Both species have very similar patterns of spur arrangement on the metafemur, but they differ in the arrangement of spurs on metatibiae (compare Fig. 6 and Fig. 1a, c in Liu et al. [20]). Both species also differ in the shape of the pronotum, which is quadrate in *O. robustus* but distinctly longer than wide in *O. cenomanianus* (compare Fig. 1a, c in Liu et al. [20] and Fig. 1a, b, and c in Yin et al. [19]). The difference is also in antennomeres 4–6, which are slender in *O. robustus* (Fig. 6a, b) but more robust in *O. cenomanianus* (Fig. 5a), and in the shape of cerci, which are slender in *O. robustus* (Fig. 6b, d) but more robust in *O. cenomanianus* (Fig. 5d, e)*.*

*Zorotypus hirsutus* is synonymized here with *Octozoros robustus* on the basis of detailed morphological comparisons between published descriptions and available fossil material (see above and Materials and Methods), which led to the finding that all diagnostic characters match and that there are actually no morphological differences between these species. Both species were described in the same year, and neither author mentioned the existence of the other of these species in the original publications, and it is obvious that the authors were unaware of the concurrent descriptions.

***Octozoros pecten*** **(Mashimo, 2019), comb. nov.**

*Zorotypus (Octozoros) pecten* Mashimo, 2019 in Mashimo et al., 2019: 566 [1].

**Comments** This species is known only from the type specimen, which is a well-preserved alate male [1]. Genus-level diagnostic characters are well observable, including the 8-segmented antennae, CuA_1_ present on the forewings, hind tibiae with two spurs in the distal third and the additional tiny apical spine developed inside the ventral surface, ctenidia present on both sides of T10, and MP present on T11. Genitalia not observable. *Octozoros pecten* is similar to *O. cenomanianus* but differs in the presence of a group of thick setae in the middle of T10.

**Genus** ***Cretozoros*** **gen. nov.**

urn:lsid:zoobank.org:act:2FC09AE9-C2E5-4898-8DB7-0B30333123FE.

(Fig. 7).

**Fig. 7:**
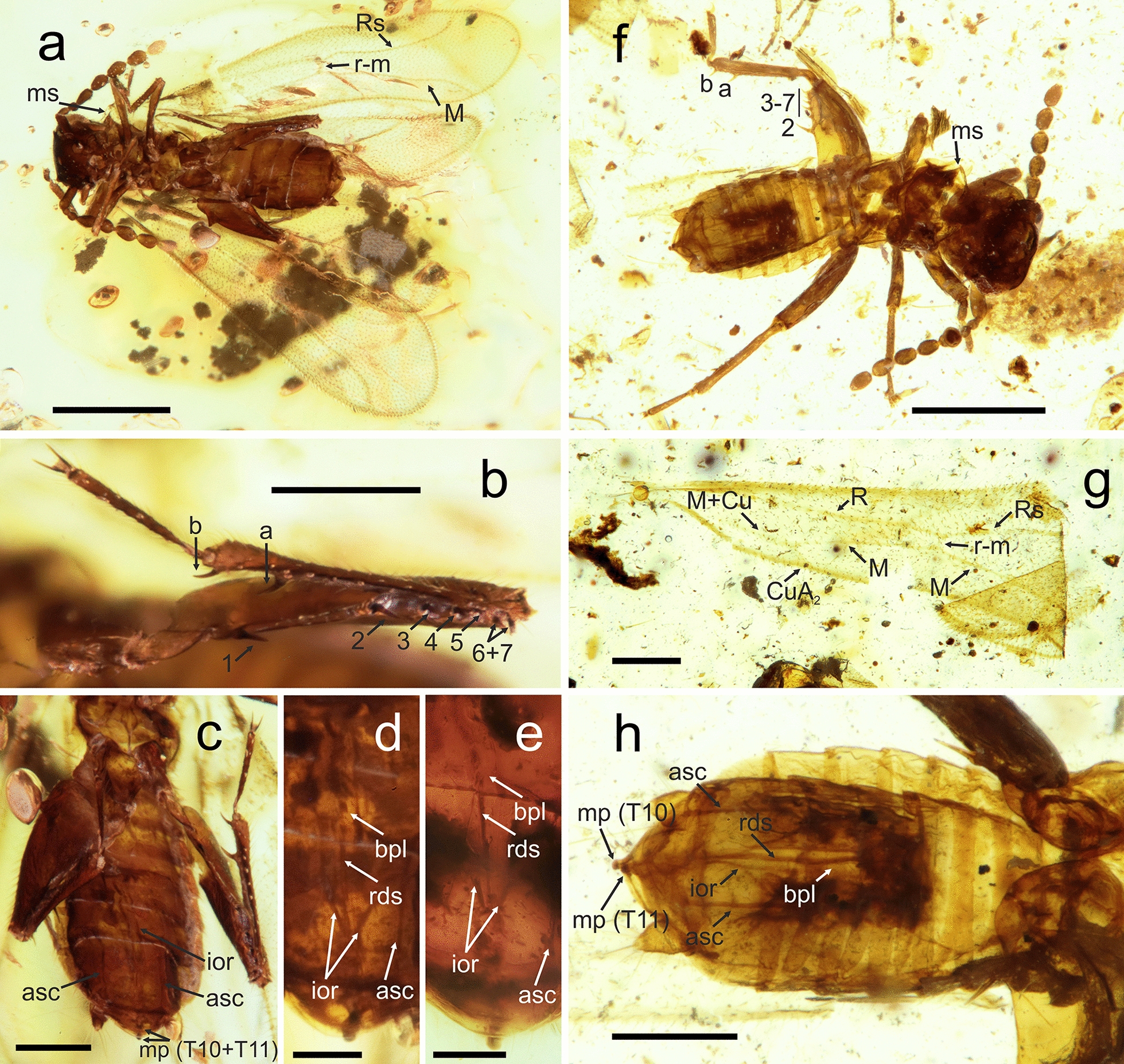
Habitus and details of *Cretozoros acanthothorax* (Engel & Grimaldi, 2002), comb. nov. **a**–**e** Specimen PK342Bu, male; **f**–**h** specimen PK347Bu, male. (**a**, **f**) General habitus. (**b**) Detail of the right metafemur and metatibia, ventral view. (**c**, **h**) Abdomen, ventral view. (**d**, **e**) Detail of male genitalia, with different focus layers and lighting, ventral view. **g** Left forewing, ventral view. Abbreviations: a, b, metatibial spurs; asc, lateral accessory sclerites; bpl, basal plate; Cu, cubitus vein; CuA, anterior cubitus vein; ior, intromittent organ; M, media vein; ms, mesonotal spine; R (Rs), radius vein; mp, median projection; rds, median rod-like sclerites; S10–S11, sternites; T10–T11, tergites; 1–7, metafemoral spurs. Scale bars: 0.5 mm (**a**, **f**), 0.2 mm (**b**, **c**, **g**, **h**), and 0.1 mm (**d**, **e**)

**Type species** *Zorotypus acanthothorax* Engel & Grimaldi, 2002, here designated.

**Etymology** The generic name refers to an exclusive occurrence in the Cretaceous period, in combination with a suffix derived from the word base of the order name. Gender masculine.

**Diagnosis** *Cretozoros* gen. nov. is characterized by the following unique combination of characters: antennae 8-segmented; forewings with reduced venation; Rs not developed; R continuing from the radial stem and not divided into R and Rs distally from r-m crossvein and continuing as R; both M and R reaching posterior wing margin near the apex; CuA_1_ absent (Fig. 3e); ventral margin of the metafemur without a furrow; hind tibiae with two spurs in distal third; and in males, ctenidia are absent on T10, central regions of T10 and T11 with short upcurved MPs (MP T11 not visible or absent in *C. pussilus* (Chen & Su, 2019)), and male genitalia symmetrical with a tongue-like anterior process encircled by the elongated intromittent organ and laterally developed rod-shaped accessory sclerites.

*Cretozoros* gen. nov. differs from *Octozoros* in the absence of CuA_1_ on forewings, ctenidia on T10, and MPs on both T10 and T11 (Tables 1 and 2). *Cretozoros* gen. nov. differs from recent *Latinozoros* in the presence of rod-shaped accessory sclerites lateral to male genitalia. The antennae of *Cretozoros* are composed of eight antennomeres in adults of both sexes, whereas the antennae of *Latinozoros* adults are always composed of nine antennomeres in adults.

**Systematic placement** *Cretozoros* gen. nov. is herein assigned to Spiralizoridae: Latinozorinae. It shares a derived wing venation pattern (absence of CuA_1_), the development of two spurs on the metatibiae, and the morphology of the male genitalia (a developed basal plate encircled by the intromittent organ) (Tables 1 and 2) with recent *Latinozoros*. *Cretozoros* gen. nov. shares an apomorphic state of the antennae (eight segments) with *Octozoros* Engel, 2003, stat. nov. and *Paleospinosus* Kaddumi, 2005, stat. restit., and we consider *Octozoros/Cretozoros/Paleospinosus* clade to be the sister group of *Latinozoros*, which has an ancestral state of this character (nine segments).

**Species included** *Cretozoros acanthothorax* (Engel & Grimaldi 2002) (= *Z. hukawngi* Chen & Su, 2019 [24], syn. nov.); *C. pusillus* (Chen & Su, 2019), comb. nov. [16].

**Distribution** Myanmar, Kachin State, Myitkyina District, Hukawng Valley, Burmese amber (Upper Cretaceous, lower Cenomanian).

***Cretozoros acanthothorax*** **(Engel & Grimaldi, 2002), comb. nov.**

*Zorotypus acanthothorax* Engel & Grimaldi, 2002: 10 [22].

 = *Zorotypus (Octozoros) hukawngi* Chen & Su, 2019: 264 [24], syn. nov.

(Fig. 7).

**Materials examined** Four adult specimens: three alate males, PK342Bu, PK347Bu, and PK340Bu, and one alate female, PK346Bu (all Burmese amber).

**Supplementary description** Based on the study of new material and detailed morphological comparisons, we find that there are no morphological differences between *Cretozoros acanthothorax* and *Zorotypus hukawngi*. Therefore, we synonymize *Z. hukawngi* with *C. acanthothorax*.

We supplement the description of *C*. *acanthothorax* with the following morphological characters, which are not observable in either the *C. acanthothorax* holotype or in the *Z. hukawngi* holotype. *Cretozoros acanthothorax* was described based on a male specimen, and *Z. hukawngi* was described based on a female specimen. Newly studied fossil material includes two males (PK347Bu and PK342Bu) and one female (PK346Bu).

**Wing venation** Wings are hyaline with dense pubescence, with a forewing length of 1.64 mm and a hindwing length of 1.36 (Fig. 7a, g). Wing venation is faint with most veins represented by fuscous lines; membrane is hyaline with scattered minute setae except infuscation forming slightly sclerotized pterostigma in the forewing; marginal setae on both the forewings and hindwings are numerous and short, and longer than setae on the membrane; and the posterior margin of the forewing has jugate setae in the middle third. Forewings show reduced venation; R continues from the radial stem and is not divided into R and Rs distally from the rs-m crossvein (in the midpoint) and continues as R; M and R reach the posterior wing margin near the wing apex, and CuA_1_ is absent. Hindwing with M + R in the anterior half; both R and M reach the wing margins; the basal third of the hindwing is not visible in any studied specimen; and Cu is absent.

**Male abdomen** T10 is smooth, without ctenidia, and central regions of T10 and T11 are distal with short peg-like MPs. Comment: MPs are short and thus not well visible in some specimens—compare Fig. 7c, d, e, and h. In the original description of *C*. *acanthothorax*, only MP T11 is depicted (Fig. 10 [22]), but this fact is not mentioned in the description itself.

**Male genitalia** Genitalia symmetrical with a minute basal part and long tongue-like anterior process (basal plate) encircled by the intromittent organ, an axis of the basal plate with median rod-like sclerites, and accessory rod-like sclerites developed laterally (Fig. 7c, d, e, h).

***Cretozoros pusillus*** **(Chen & Su, 2019), comb. nov.**

*Zorotypus (Octozoros) pusillus* Chen & Su, 2019: 556 [16].

**Comments** This species is known only from the type series, which is composed of alate males (holotype) and alate females (paratype) embedded in the copula position in one amber piece [16]. Genus-level diagnostic characters are partly observable, including the 8-segmented antennae, the hind tibiae with two spurs developed in the distal third, and the ctenidia not developed. MP is visible on T10, but the presence/absence of the projection on T11 cannot be evaluated because of the position of the fossil. The male genital is in an everted position and is symmetrical; however, the morphological details are not recognizable. Lateral rod-shaped accessory sclerites are visible. The arrangement of spines on the ventral surface of the metafemur is species-specific.

**Genus** ***Paleospinosus*** **Kaddumi, 2005, stat. restit.**

*Palaeospinosus* Kaddumi, 2007: 218 [17].

**Type species** *Paleospinosus hudae* Kaddumi, 2005, by original designation [25].

**Updated diagnosis** *Paleospinosus* is characterized by the following combination of characters: antennae 8-segmented; anterolateral spines on the mesonotum absent; ventral margin of the metafemur with a deep furrow that extends from the apex to the middle; hind tibiae with two spurs in the distal third; in males, T10 with two clusters of thick setae on both sides of the posterior margin; one long and thin upcurved MP developed, although it is not clear if this is a projection of T10 or T11; and the posterior edge of S8 broadly emarginate with a peg-like projection in the middle. Wing venation is not distinctly recognizable in the holotype of the type species and therefore cannot be used for diagnosis.

Within Latinozorinae, males of *Paleospinosus* differ from those of other genera in the following ways: from *Cretozoros* gen. nov. in the presence of two clumps of thick setae on both sides of the T10 posterior margin, one developed long upcurved MP, and the posterior edge of S8 broadly emarginate; from *Octozoros* in the absence of ctenidia on T10 and broadly emarginate posterior edge of S8; and from recent *Latinozoros* in terms of the number of MPs in the male abdomen (while *Paleospinosus* has only one MP, *Latinozoros* has MPs on both T10 and T11) and the number of antennomeres (eight in *Paleospinosus*, nine in *Latinozoros*).

**Systematic placement** The genus *Paleospinosus* was synonymized with *Octozoros* Engel, 2003 (by that time a subgenus of *Zorotypus*) by Engel [26]. *Paleospinosus* is herein removed from that synonymy and reinstated as a valid genus in Spiralizoridae: Latinozorinae on the basis of the arrangement of metatibia spurs. *Paleospinosus* differs from *Octozoros* gen. nov. and *Cretozoros* gen. nov. deeply furrowed metafemur (Tables 1 and 2). The wing venation of the holotype of *Paleospinosus* is not clearly identifiable, but the reduction of some veins (Rs and possibly CuA) distinguishes this genus from *Cretozoros* gen. nov., which has fully developed venation. *Paleospinosus* shares an apomorphic state of the antennae (eight segments) with *Octozoros* and *Cretozoros*, and we consider *Octozoros/Cretozoros/Paleospinosus* clade to be the sister group of *Latinozoros*, which has an ancestral state of this character (nine segments).

**Species included** *Paleospinosus hudae* Kaddumi, 2005.

**Distribution** Jordan, Zarqa River Basin (Lower Cretaceous, Albian).

***Paleospinosus hudae*** **Kaddumi, 2005**

*Palaeospinosus hudae* Kaddumi, 2007: 218 [17].

**Comments** This species is known only from the holotype, which is an alate male [17]. The specimen is well preserved and allows us to observe important diagnostic characters, although the wing venation is only partly recognizable. Kaddumi [17] characterized the venation as reduced, but Engel [26] described it as a typical zorapteran venation. Although the basal part of the wings is not easily observable in the holotype, the reduction in Rs and CuA is recognizable. The genitalia are not observable.

**Mesozoic Zoraptera** **incertae sedis**

***Zorotypus cretatus*** **Engel & Grimaldi, 2002**

*Zorotypus cretatus* Engel & Grimaldi, 2002: 4 [22].

**Distribution** Myanmar, Kachin State, Myitkyina District, Hukawng Valley, Burmese amber (Upper Cretaceous, lower Cenomanian).

**Comments** This species is known only from apterous males [22]. *Zorotypus cretatus* could not be classified because of poor fossil preservation and a lack of observable diagnostic characters.

***Zorotypus dilaticeps*** **Yin, Cai, Huang & Engel, 2018**

*Zorotypus dilaticeps* Yin, Cai, Huang & Engel, 2018: 127 [21].

**Distribution** Myanmar, Kachin State, Myitkyina District, Hukawng Valley, Burmese amber (Upper Cretaceous, lower Cenomanian).

**Comments** *Zorotypus dilaticeps* is known only from an apterous (dealate) female type specimen [21]. It is a large species (3.9 mm) that can be distinguished from all other extinct and recent Zoraptera by its distinctive head morphology and the spination of the metafemur and metatibia. The inner margin of the metatibia is armed with six acute spines and seven spine-like setae. Such a spination is unique among fossil Zorapterans and does not allow classification to any extant genus; nevertheless, such an arrangement does not need to be necessary for a genus-diagnostic character. Within recent groups of Zoraptera, a similar example of a secondary spination of metatibia appears in *Brazilozoros huxleyi* (Bolívar y Pieltain & Coronado, 1963), although other representatives of the genus do not have any spination [2, 14, 27]. Because the holotype specimen is a dealate female, the placement of this taxon in a higher classification is not possible at the moment.

## Identification key to the fossil genera of Zoraptera (males only)

1 Antennae composed of eight antennomeres; metatibia with two robust spurs and an additional tiny apical spine on the inside of the ventral surface present or absent; genitalia symmetrical ……………………2

- Antennae composed of nine antennomeres; metatibia with three robust spurs, additional apical spine on the inside of the ventral surface not developed; genitalia asymmetrical …………………4

2 Ventral margin of the metafemur with a deep longitudinal furrow; posterior edge of S8 broadly emarginate with a peg-like projection in the middle … *Paleospinosus* Kaddumi, 2005, stat. restit.

- Ventral margin of the metafemur entire or with only shallow longitudinal furrow; posterior edge of S8 entire …………………………3

3 Forewing with CuA_1_ absent; ctenidium on T10 absent, MPs on both T10 and T11, genitalia with laterally developed rod-shaped accessory sclerites … *Cretozoros* gen. nov.

- Forewing with CuA_1_ developed; ctenidium on T10 present; MP only on T11, genitalia without laterally developed rod-shaped accessory sclerites … *Octozoros* Engel, 2003, stat. nov.

4 Conical MPs on T10 and T11………… *Burmazoros* gen. nov.

- Long, thin and procurved mating hook only on T11 … *Xenozorotypus* Engel & Grimaldi, 2002

## Discussion

This study presents the first critical revision of the previously described fossil species of Zoraptera from the Mesozoic, leading to their classification (Tables 3 and 4) based on the recently established system of valid diagnostic characters for recent species [13]. Currently, 11 Mesozoic Zoraptera species are recognized (Table 4). On the basis of our morphological examination, nine species can be assigned to five genera, i.e., *Burmazoros* gen. nov., *Cretozoros* gen. nov., *Octozoros* stat. nov., *Paleospinosus* Kaddumi, 2005, stat. restit., and *Xenozorotypus*. Owing to the absence of males with visible genitalia, the poor preservation of fossils, and some characters that are difficult to homologize, we provisionally retain the remaining two Mesozoic species in the genus *Zorotypus* (*Z. cretatus* and *Z. dilaticeps*, which are classified as 'incertae sedis'; see Table 4).

The current Zoraptera systematics is based mainly on male genitalia characters [13]. The principal family diagnostic character is the symmetry/asymmetry of the male copulatory organ, with representatives of Zorotypidae having asymmetrical genitalia without a developed basal plate (plesiomorphy), while Spiralizoridae have symmetrical genitalia with a developed basal plate (apomorphy) (Fig. 2). The current division of both families into subfamilies also reflects the morphology of the genitalia. The number and degree of symmetry of the sclerites is a diagnostic character for Zorotypinae vs. Spermozorinae, and the state of intromittent organ is a diagnostic character for Latinozorinae vs. Spiralizorinae [13]. Male genitalia have not yet been properly studied and described in fossil Zoraptera, and these can be observed only in two published specimens, each assigned to a different genus (*Cretozoros pusillus*, *Burmazoros denticulatus*). In *C. pusillus*, the everted male genitalia are difficult to homologize [16], whereas in *B. denticulatus*, male genitalia are partly visible in the holotype; however, they were not described by the authors [19]. Based on Fig. 2 [19], the genitalia appear to be asymmetrical, the basal plate and elongated intromittent organ are apparently absent. Within the extensively studied material of Mesozoic Zoraptera, we managed to find three male specimens with observable genitalia. This enabled us to homologize their genitalia with those of recent groups and, subsequently, the inclusion of most Mesozoic species in the current suprageneric classification of the order. Kočárek et al. [13] reported a correlation between genital characteristics and the number of spurs on the metatibia. These characteristics were used for diagnosing genera. In addition to the characteristics of the copulatory organs and metatibia, the following characteristics were also used for the generic diagnoses: variable characteristics on the abdominal tergites (MPs on T10 and T11), the presence of ctenidia on T10 and several characteristics of the forewings and hindwings.

To evaluate the phylogenetic relationships of the newly identified fossil taxa of Zoraptera, it was necessary to estimate the evolutionary histories of selected morphological features. Ancestral character state reconstruction (ASR) was performed on recent representatives of Zoraptera using phylogenetic analyses published by Kočárek et al. [13], who evaluated characters that are also observable in fossils (Fig. 1). ASR was performed on four characters related to reproduction: three related to the morphology of male genitalia and one related to MPs on male abdominal tergites T10 and T11. Furthermore, three states related to the development of spurs on the metatibia and one character related to venation on the wings (the absence of CuA_1_) were evaluated. We demonstrated that all these characters bear a phylogenetic signal. Previous studies have demonstrated the correlation between the number of metatibial spurs and genital characters in recent representatives [13].

Although male copulatory organs are morphologically complex and can be used to assess more detailed morphological features of individual sclerites [28, 29], we focused only on three features: symmetry, development of the basal plate, and development of the elongated intromittent organ. First, there is still no consensus regarding the structural homology of genitalia in Zoraptera [9, 28, 29], with scientists using different terminology on the basis of different interpretations of the literature. A reliable assessment of homologous features awaits resolution [9, 30]. Second, the purpose of the analyses was to evaluate the characteristics of the fossil specimens, for which detailed microscopic evaluation of individual sclerites is impossible. Internal structures are only exceptionally preserved in amber, and even when they are partially preserved, a detailed examination of their microstructure is impossible. In terms of symmetry, we estimate that the ancestral state of Zoraptera was probably symmetrical (Fig. 1), a conclusion also reached by the authors of a previous study [9]. Asymmetrical genitalia configurations have been documented in various Polyneoptera groups [31], but those are likely to be derived states, with the symmetrical state being considered ancestral for Polyneoptera [30]. According to our analyses, the presence of the elongated intromittent organ is probably ancestral. The elongated intromittent organ is apomorphic for Spiralizoridae, although it secondarily missing in the Neotropical genus *Brazilozoros* [2] and the Oriental genus *Aspiralizoros* [32]. Our results contradict those of Matsumura et al. [9], who conducted an ancestral analysis evaluating the absence of an elongation of intromittent organ as the ancestral state. However, we believe that their analysis was influenced by the taxa included, with those with probable secondary absence (the genus *Brazilozoros*) being more numerous at the expense of other zorapteran groups. The ancestral type of the elongated intromittent organ remains unclear (Fig. 1d). Nevertheless, we consider the vertically coiled intromittent organ to be synapomorphy of Spiralizorinae (Figs. 1, 2 and 4). In accordance with the results of Matsumura et al. [9], the presence of the basal plate was evaluated as the ancestral state (Fig. 1b), with an apomorphic disappearance in Zorotypidae in accordance with the evolutionary rearrangement of the genitalia. The presence or absence of this character is correlated with genital symmetry and the development of the intromittent organ.

The hind tibiae of Zoraptera bear conspicuous spurs, while the presence or absence and number of these spurs were evaluated as diagnostic characters at the subfamily level and correlated with both molecular phylogeny and male genitalia characters [13]; this correlation has been verified in subsequent studies [2, 4, 7, 32–34]. The absence of spurs was evaluated as the ancestral state, while the presence of three spurs (Zorotypinae) and two spurs (Latinozorinae) were considered synapomorphies of these subfamilies (Fig. 1g). This feature is very important for classifying and identifying Zoraptera fossils, as it is a nongenital character that can be observed in females and poorly preserved fossils. In the genera *Octozoros* Engel, 2003, stat. nov., *Burmazoros* gen. nov. and *Cretozoros* gen. nov., the number of spurs on the metatibiae fully correlates with the morphology of the male genitalia, as documented in the current system, constructed using a molecular phylogenetic approach. This approach can also be used to classify species without observable genitalia. The three newly defined (or reinstated) genera, *Octozoros*, *Cretozoros* and *Paleospinosus*, include individuals with two spurs on the metatibiae (Table 1). Since the characteristics of their genitalia are similar to those of the Latinozorinae subfamily, we classified them into this group (Table 4). The asymmetrical genitalia in the newly erected genus *Burmazoros* correlate with three spurs on the metatibiae, as in the recent Zorotypinae; therefore, we classify it into this group. The same applies to *Xenozorotypus burmiticus*, which is provisionally assigned to the same subfamily on the basis of metatibia morphology, despite the unknown morphology of the male copulatory organ (Table 4).

Although we identified synapomorphies in both the forewings and hindwings, the phylogenetic significance of wing venation remains questionable. Kukalova-Peck and Peck [35] proposed the first generic classification of recent Zoraptera based on wing venation. However, this was not accepted by the scientific community [22] because of the high degree of similarity between species and variability within species. Since then, no author has assessed the importance of wing venation for phylogenetic reconstruction [9, 13]. One of the few apomorphies identified in our ancestral analysis, which was also defined by Kukalova-Peck and Peck [35], is the absence of the CuA_1_ vein in the genus *Latinozoros* (Fig. 3b). This absence was also found in the newly defined genus *Cretozoros* gen. nov. This vein is present in the genus *Octozoros* Engel, 2003, stat. nov. Conversely, *Burmazoros* gen. nov. exhibit a pattern of forewing venation with strongly reduced veins R, M, and Cu (Fig. 3 and Table 2). We hypothesize that a secondary reduction of veins occurred in these cases. We suppose the clade *Paleospinosus*/*Octozoros* sister to *Cretozoros* defined by absence of CuA_1_ and Rs on the forewings (see Fig. 4 and Table 2). Anyway, the poor preservation of the wings and genitals in the only known fossil of *Paleospinosus* Kaddumi, 2005 does not permit the certainty required for evaluation of these characters and thus relationships between the genera *Cretozoros*, *Paleospinosus* and *Octozoros* staying unclear. Conversely, the genus *Xenozorotypus* has a unique arrangement of veins on the hindwing (the presence of the M_3+4_ vein), which we suppose to be an apomorphy (Fig. 3i and Table 2). We believe that wing venation has potential in both phylogenetic reconstruction and taxonomy and that a new, extensive analysis in light of recently established molecular phylogenetic relationships is highly desirable.

For some recent genera and seemingly also for some fossil genera, a diagnostic feature is the presence of MPs on the posterior parts of abdominal tergites T10 and T11 in males [13]. Two states of this character are known in fossil Zoraptera: the presence of MPs on both T10 and T11, and the presence of MP only on T11 (Table 1). It has been recently shown that this character remains consistent across all species within certain higher taxonomic groups (e.g., all recent Spiralizorinae species have an MP on T11 only, whereas recent Latinozorinae and Spermozorinae species have MPs on both T10 and T11). However, some recent *Zorotypus* species have a MP on T10, T11 or both [4, 9, 13]. According to our analysis, the ancestral state in Zoraptera was probably T10 + T11 present (Fig. 1e). Among fossil species, we also observed variability in this character state within the same subfamily, although it appeared stable within defined genera (Fig. 4 and Table 2).

From an evolutionary perspective, an interesting feature is the number of antennomeres. All recent Zoraptera species have nine antennomeres in the adult state [9, 13]. However, a state with eight antennomeres is predominant in the Mesozoic fossils, with eight out of the eleven distinguished species showing this state (Table 2). On this basis, the fossil subgenus *Octozoros* Engel, 2003, stat. nov. was described [18]. As part of our revision of the system, this subgenus was elevated to a genus; however, the species originally included in this subgenus are now classified into three genera: *Octozoros* Engel, 2003, stat. nov., *Cretozoros* sp. nov., and *Paleospinosus* Kaddumi, 2005, stat. restit. (Spiralizoridae: Latinozorinae). We consider the reduction of antennomeres to eight to be an apomorphic state, and we define a clade comprising these three genera as the sister group of the recent *Latinozoros* with nine antennomeres (Fig. 4).

An important aspect of studying fossils in amber, not only those of Zoraptera, is the need to carefully compare the morphology of the examined material with that of already described species. This is because newly described species are often just additional specimens of previously described species. This is due to the different levels of preservation and positions of the fossils, which result in different levels of structural observability, as well as secondary changes in shape caused by compression [36]. Another significant aspect is the usual observation of only one sex, which may exhibit sex-specific characteristics. In the case of Zoraptera, the situation is further complicated by the presence of winged and wingless specimens, as well as by the thin cuticle of their bodies, which deforms easily in amber. At the species level, the distribution pattern of spurs on the metafemur seems to be a useful diagnostic character. However, its usefulness for higher-level classification is minimal, as demonstrated by a recent phylogenetic study of Zoraptera [13]. We recommend adhering to the approach of considering examined individuals to be the same species if they have the same pattern of spines on the metafemur and metatibia as the compared species, assuming the specimen does not show any other prominent apomorphy (e.g., wing venation or spines on thoracic segments). Caution is especially needed when assessing the shape and length of cerci and antennomeres, as these are both susceptible to deformation and can appear different even in specimens of the same species. To correctly assess these characters, it is recommended that several specimens be compared.

## Materials and methods

We critically reviewed all the relevant literature on the fossil Mesozoic Zoraptera to assess their systematic placement. In addition to the already published information, we studied in detail several newly reported specimens of the most critical taxa. The studied material is preserved in fossil resin originally produced by representatives of the tree family Araucariaceae [37]. The studied pieces of amber were found in the surroundings of Tanai Village (26° 21′ N, 96° 43′ E) in the Hukawng Valley of Myanmar [38–40], but the precise mining sites are unknown because of the mixing of samples obtained from local miners. The deposits in Tanai Village have been investigated and dated in detail by Cruickshank & Ko [39] and Shi et al. [41]; the age has been estimated to be ca. 99 Ma (98.8 ± 0.6; lower Cenomanian) on the basis of U–Pb dating of zircons from the volcaniclastic matrix of the amber [41]. The studied specimens were collected before 2017 and were legally exported from Myanmar (see discussion in Haug et al. [42]). The amber pieces containing the samples were ground, polished, and then examined with a Leica Z16 APO macroscope (Leica Microsystems, Wetzlar, Germany) equipped with a Canon 6D Mark II camera (Canon Inc., Tokyo, Japan). Micrographs of 20 to 30 focal layers of each specimen were combined with Helicon Focus software (Helicon Soft Ltd., Kharkiv, Ukraine) and finally processed with Adobe Photoshop CS6 Extended v13 (Adobe Inc., San Jose, California).

In this study, the following fossil samples deposited at the Department of Biology of the University of Ostrava, Czech Republic, were examined: *Octozoros cenomanianus* (Yin, Cai & Huang, 2018), comb. nov.: PK344Bu (alate male); *O. robustus* (Liu, Zhang, Cai & Li, 2018), comb. nov.: PK348Bu (alate female), PK343Bu (apterous male), PK336Bu (apterous female), and PK164Bu (alate female); *Cretozoros acanthothorax* (Engel & Grimaldi, 2002), comb. nov.: PK342Bu (alate male), PK347Bu (alate male), PK340Bu (alate male), and PK346Bu (alate female). Illustrations of subfamily diagnostic characters were adopted from Kočárek et al. [13] and supplemented by a microphotograph of the hind leg of *Spermozoros weiweii* (Wang, Li & Cai, 2016), which was collected in Brunei Darussalam (Ulu Temburong NP, Sungai Apan II, N 4° 33.22950′ E 115° 10.59277′, 12.–19.ii.2015, P. Kočárek & I. Horká leg). The higher classification and morphological terminology of Zoraptera follows that of Kočárek et al. [13]. The geological periods and epochs follow the International Chronostratigraphic Chart v2023/09 [43].

To assess the phylogenetic relationships of fossil Zoraptera and classify them into a system based on recent representatives, we examined the evolution of observable characters in the fossil record. Specifically, we examined the following characteristics in both recent and fossil species: (1) the symmetry of male genitalia; (2) the absence or presence of a basal plate; (3) the absence or presence of an elongated intromittent organ; (4) the type of intromittent organ; (5) the development and number of spurs on the metatibia; (6) the development of the abdominal tergite T10 and T11 MPs; (7) the development of the forewings, including CuA_1_ and CuA_2_; (8) the spiralization of the vertically coiled intromittent organ; (9) the development of the hindwing M; (10) the absence or presence of the forewing vein Rs; (11) the number of antennomeres; and (12) metafemur furrowing.

To evaluate the evolutionary pathways of selected morphological characters and assess the phylogenetic relationships of fossil species, ancestral character state reconstruction (ASR) was performed using Mesquite v4.01 [44]. Maximum likelihood (ML) analysis of ancestral states was based on the ML topology of the three-marker tree published by Kočárek et al. [13], taking into account branch lengths and using the Markov k-state one-parameter model. Traits 1–7 (see above) were included in the ASR. Traits and their derived states that occur only in fossil representatives or in one terminal clade (traits 8–12) were not included in the ASR but were used for the genus and species diagnoses of fossil representatives. The morphological character states of extant and extinct Zoraptera are listed in Supplementary Table S1 and Tables 1 and 2, respectively.

The ASR analysis was carried out with and without outgroups, with the results being identical for all separate characters and differing only slightly in terms of the probability of the ancestral state for some characters; thus, only the results without outgroups are presented in Fig. 1. Relevant information about morphological character states was obtained from the literature (see Kočárek et al. [13]) and was based on microscopic studies of the representatives included in the analyses. Relevant information for the outgroup was obtained from Steinmann [45–47].

## Conclusions

Our results led to the proposal of a generic classification of Mesozoic Zoraptera. We describe two new genera (*Burmazoros* gen. nov. and *Cretozoros* gen. nov.), reinstate *Paleospinosus* Kaddumi, 2005, stat. restit., as a distinct genus, and elevate *Octozoros* Engel, 2003, to the genus level. Overall, nine out of the 11 currently recognized species of Mesozoic Zoraptera were classified. *Zorotypus hukawngi* Chen & Su, 2019 is a synonym of *Cretozoros acanthothorax* (Engel & Grimaldi, 2002) comb. nov., and *Zorotypus hirsutus* Mashimo, 2018 is a synonym of *Octozoros robustus* (Liu, Zhang, Cai & Li, 2018) stat. restit., comb. nov., which is simultaneously restored from synonymy with *Octozoros cenomanianus* (Yin, Cai & Huang, 2018), comb. nov.

Based on the shared polarized morphological characters and comparative morphology of other structures, we present a phylogenetic scheme for the evolution of the Zoraptera and propose the systematic positions of newly established fossil taxa. *Cretozoros* gen. nov., *Paleospinosus* Kaddumi, 2005, stat. restit. and *Octozoros* Engel, 2003, stat. nov. are classified in Spiralizoridae: Latinozorinae, while *Burmazoros* gen. nov. and *Xenozorotypus* are classified in Zorotypidae: Zorotypinae. Based on synapomorphies, we propose that the fossil species of *Cretozoros, Octozoros* and *Paleospinosus* form a clade sister to the recent species of *Latinozoros*, and that the fossil genus *Xenozorotypus* is a sister to the clade composed of fossil *Burmazoros* + recent *Zorotypus*/*Usazoros*. Classifying Mesozoic Zoraptera within the modern system enables us to better understand the diversity of their internal lineages during the early evolution of this enigmatic insect order.

## Supplementary Information

Additional file1 (PDF 169 KB)

## Data Availability

All the data generated or analyzed during this study are included in this published article.

## Abbreviations

Cu: Cubitus vein
CuA: Anterior cubitus vein
M: Media vein
MP: Median projection
R: Radius vein
S: Abdominal sternite
T: Abdominal tergite
