# Supplementary material for: Morphology of male genitalia, legs, and wing venation reveals the classification of Mesozoic Zoraptera (Insecta)
Source: Front Zool. 2026 Feb 7;23:8. doi: 10.1186/s12983-025-00595-x (PMC12977874; doi:10.1186/s12983-025-00595-x)
Supplement: Supplementary file 1 — Additional file1 (PDF 169 KB) [file 12983_2025_595_MOESM1_ESM.pdf]

Supplementary Table S1. Character matrix of selected morphological traits of Zoraptera used for ancestral state analysis in Mesquite. Detailed information on the analyzed taxa is provided by Kočárek et al. [13].

Taxon	Symmetry of male genitalia	Presence of basal plate	Elongation of intromittent organ	State of intromittent organ	T10 & T11 median projections	Forewing CuA_1 & CuA_2	Hind tibia spurs
Brazilozoros huxleyi	0	1	0	0	2	0	0
Brazilozoros weidneri	0	1	0	0	2	?	0
Centrozoros neotropicus	0	1	1	1	2	0	0
Centrozoros sp. 1	?	?	?	?	?	0	0
Latinozoros cacaoensis	0	1	1	2	1	1	1
Latinozoros gimmeli	0	1	1	2	1	1	1
Scapulizoros novobrittanicus	0	1	1	1	2	?	0
Spermozoros asymmetricus	1	0	0	0	1	0	0
Spermozoros impolitus	1	0	0	0	1	0	0
Spermozoros medoensis	1	0	0	0	1	0	0
Spermozoros weiweii	1	0	0	0	1	0	0
Spiralizoros caudelli	0	1	1	1	2	0	0
Spiralizoros cervicornis	0	1	1	1	2	0	0
Spiralizoros hainanensis	0	1	1	1	2	?	0
Spiralizoros sp.	0	1	1	1	2	?	0
Spiralizoros sp. 2	0	1	1	1	2	0	0
Spiralizoros sp. 3	0	1	1	1	2	0	0
Spiralizoros sp. 4	0	1	1	1	2	?	0
Usazoros hubbardi	1	0	0	0	1	0	2
Zorotypus delamarei	1	0	0	0	1	0	2
**Outgroup**							
Anechura bipunctata	0	–	1	–	0	–	0
Cranopygia sp.	0	–	1	–	0	–	0
Euborellia arcanum	0	–	1	–	0	–	0
Forficula auricularia	0	–	1	–	0	–	0
Chelidurella acanthopygia	0	–	1	–	0	–	0
Nannopygia sp.	0	–	1	–	0	–	0
Pyragropsis thoracica	0	–	0	–	0	–	0
	**Character states**	**Character states**	**Character states**	**Character states**	**Character states**	**Character states**	**Character states**
	0 - symmetric	0 - absent	0 - not elongated or absent	0 - not elongated or absent	0 - absent	0 - CuA_1 & CuA_2 present	0 - absent
	1 - asymmetric	1 - present	1 - elongated	1 - vertically coiled	1 - T10 + T11 present	1 - CuA_1 absent	1 - two spurs
	? - unknown	? - unknown	? - unknown	2 - horizontally coiled	2 - T11 only present	? - unknown	2 - three spurs
		- inapplicable status		? - unknown	? - unknown	- inapplicable status	
				- inapplicable status			
